# Recent studies on myricetin and its biological and pharmacological activities

**DOI:** 10.17179/excli2023-6571

**Published:** 2023-11-28

**Authors:** Priscilla Nadalin, Jae Kwang Kim, Sang Un Park

**Affiliations:** 1 Department of Crop Science, Chungnam National University, 99 Daehak-ro, Yuseong-gu, Daejeon, 34134, Korea; 2 Division of Life Sciences and Convergence Research Center for Insect Vectors, College of Life Sciences and Bioengineering, Incheon National University, Incheon 22012, Korea

## ⁯

Flavonoids are compounds characterized by a 15-carbon skeleton structure (also abbreviated as C6-C3-C6) containing two aromatic ring systems (A and B rings) and a heterocyclic ring (C) (Kumar and Pandey, 2013[37]; Dias et al., 2021[19]). Among these, flavonols have an unsaturated C ring at the C2-C3 position, which is hydroxylated at C3 and oxidized at C4. The main flavonols are myricetin (MYR), quercetin, kaempferol, isorhamnetin, and fisetin (Kim et al., 2006[35]; Spiegel et al., 2020[61]). 

MYR (3,3′,4′,5,5′,7-hexahydroxyflavone) is a flavonol (Ong and Khoo, 1997[48]) derived from the parent compound taxifolin, which is turned into the (+)-dihydromyricetin intermediate and can be further chemically modified to produce laricitrin and then syringetin, both molecules in the flavonol class of flavonoids (Flamini et al., 2013[21]).

MYR is present in various fruits, vegetables, tea, berries and red wine. MYR's augmented biological activity in comparison with other flavonols is due to the pyrogallol B-ring, and the more hydroxylated structure. MYR is found in the Myricaceae, Anacardiaceae, Polygonaceae, Pinaceae and Primulaceae families (Gupta et al., 2020[24]; Taheri et al., 2020[65]). MYR displays an array of biological and pharmacological activities such as antioxidant, anti-amyloidogenic, antibacterial, antiviral, antidiabetic, anticancer, anti-inflammatory, anti-epileptic and anti-ulcer (Imran et al., 2021[28]; Pluta et al., 2021[52]; Agraharam et al., 2022[1]; Javed et al., 2022[30]). Because of such a range of properties, MYR has been the object of great attention in recent years for its uses in the pharmaceutical, food, and cosmetic industries. The present letter summarizes recent key research on the biological and pharmacological properties of MYR (Table 1(Tab. 1); References in Table 1: Ahmad et al., 2022[2]; Akhtar et al., 2020[3]; Alidadi et al., 2022[4]; Alqarni et al., 2022[5]; Aminzadeh and Bashiri, 2020[6]; Aminzadeh and Salarinejad, 2021[8]; Aminzadeh et al., 2023[7]; Anwar et al., 2022[9]; Arafah et al., 2022[10]; Bai et al., 2021[11]; Berköz et al., 2020[12]; Chen et al., 2021[13], 2023[14]; Choi et al., 2021[15]; Dai et al., 2021[16]; Dang et al., 2023[17]; Deng et al., 2021[18]; Dubey et al., 2020[20]; Gao et al., 2023[22]; Gaspar et al., 2020[23]; Han et al., 2022[25]; Huang et al., 2020[26]; Ikeji et al., 2023[27]; Jang et al., 2020[29]; Ji et al., 2022[31]; Jing et al., 2021[32]; Kan et al., 2021[33]; Kang et al., 2020[34]; Kimura et al., 2021[36]; Lalitha et al., 2020[38]; Li et al., 2022[39], 2023[40]; Lin et al., 2020[41]; Liu et al., 2022[42], 2023[43]; Lv et al., 2021[44]; Ma et al., 2021[45]; Nafee et al., 2020[46]; Nie et al., 2023[47]; Pan et al., 2023[49]; Park et al., 2021[50]; Peng et al., 2022[51]; Prajapati et al., 2020[53]; Qu et al., 2020[54]; Rajendran et al., 2021[55]; Rostami et al., 2023[56]; Salimi et al., 2021[57]; Sharma et al., 2023[58]; Soleimani and Sajedi, 2020[59]; Song et al., 2021[60]; Sun et al., 2021[62]; Sur and Lee, 2022[63][64]; Wang et al., 2020[67], 2022[66], 2023[68]; Xiao et al., 2021[69]; Xu et al., 2022[70]; Yang et al., 2022[71]; Yao et al., 2022[72]; Ying et al., 2020[73]; Zhang et al., 2020[74]; Zhao et al., 2022[75]; Zhu et al., 2020[76]).

EXCLI Journal 2023;22:1223-1231 – ISSN 1611-2156
Received: September 08, 2023, accepted: November 14, 2023, published: November 28, 2023

| Key findings | Reference |
|---|---|
| MYR can lower the fibril load acting as a potent superoxide dismutase 1 (SOD1) aggregation inhibitor. MYR's structure can be a useful reference for the design of more powerful amyotrophic lateral sclerosis inhibitors that prevent the condition and reverse its symptoms. | Sharma et al., 2023 |
| MYR relieves osteomyelitis by preventing biofilm formation. This activity is due to MYR inhibiting osteoblast growth markers alkaline phosphatase, osteopontin and type-I collagen via the Toll-like receptor-2 (TLR2) and mitogen-activated protein kinase (MAPK) pathways. In silico studies indicate that MAPK may be a potential MYR-binding protein. | Gao et al., 2023 |
| MYR prevents HCoV-229E and SARS-CoV-2 replication ***in vitro***, deactivates SARS-CoV-2 virus entry facilitators, and eases inflammation via the RIPK1/NF-κB pathway. This indicates that MYR possesses the potential to become a therapeutic agent to treat COVID-19. | Pan et al., 2023 |
| MYR has proved effective against experimental Alzheimer's disease (AD), suggesting its mechanism may be related to the inhibition of mitochondrial dysfunction, activation of the NLRP3 inflammasome, and neuroinflammation induced by the P38 MAPK pathway. Therefore, MYR could be an interesting drug candidate for AD treatment. | Liu et al., 2023 |
| MYR exerts cardioprotective activity on cardiac injury caused by high-intensity exercise by downregulating PTGS2 and MAOB and upregulating MAP2K1 and EGFR, while controlling the complicated myocardial metabolic network. | Li et al., 2023 |
| MYR can ease oxidative stress and apoptosis by targeting the nuclear factor erythroid 2–related factor 2 (NRF2) signallng pathway, hence exerting a therapeutic action on hypoxic-ischemic injury. This implies that MYR could become a future hypoxic-ischemic encephalopathy treatment. | Chen et al., 2023 |
| MYR acts as an exogenous ligand for leukocyte mono-immunoglobulin-like receptor 3 (CD300f), thus downregulating MAS-related G protein-coupled receptor X2 (MRGPRX2)-mediated mast cells (MC) activation via CD300f. Therefore, MYR's activity prevents MC degranulation and pseudo-allergic reactions. | Dang et al., 2023 |
| MYR can partly prevent arsenic-induced cardiac toxicity by lowering the oxidative stress and rehabilitating the antioxidant system. | Aminzadeh et al., 2023 |
| MYR blocks the myocardium autoimmune response and monocyte chemoattractant protein-1 (MCP-1) expression in cardiomyocytes. These results imply that MYR can alleviate autoimmune myocarditis via modulation of the immune response and the expression of MCP-1. Hence, MYR could be an interesting candidate drug to treat autoimmune myocarditis. | Nie et al., 2023 |
| MYR exhibits anti-inflammatory and antioxidant properties in atrazine (ATZ)-exposed rats by alleviating ATZ-mediated functional changes in their reproductive axis. MYR as a dietary supplement may be a valid chemoprotective agent against ATZ-induced reproductive dysfunction. | Ikeji et al., 2023 |
| MYR can prevent acute liver failure by lowering inflammation and oxidative stress via NRF2 signaling. Therefore, MYR may be a promising agent to prevent liver damage. | Wang et al., 2023 |
| MYR displays an hepatoprotective activity which may be attributed to its inhibition of oxidative, inflammatory, and apoptotic factors and promotion of antioxidants, in addition to its partial regulation of sirtuin 1 and the autophagic pathway. | Rostami et al., 2023 |
| MYR may promote antioxidant activity and lower inflammation, lipotoxicity, and endoplasmic reticulum (ER) stress. These activities may be effective in reducing triacylglycerol accumulation in hepatocytes. | Yang et al., 2022 |
| MYR ameliorates memory impairment due to its anti-inflammatory activity and regulation of the brain-derived neurotropic factor (BDNF) expression. MYR may be a promising agent to protect cognitive functions from sleep deprivation. | Sur and Lee, 2022b |

EXCLI Journal 2023;22:1223-1231 – ISSN 1611-2156
Received: September 08, 2023, accepted: November 14, 2023, published: November 28, 2023

| Key findings | Reference |
|---|---|
| MYR strongly inhibits the human glutathione transferase A1-1 (hGSTA1-1) and as such it can be employed to develop natural, safe, and effective cancer chemosensitizers that target glutathione S-transferases. | Alqarni et al., 2022 |
| MYR eased the formaldehyde-enhanced Warburg effect in cancer cells by inhibiting hypoxia-inducible factor 1 subunit alpha (HIF-1α). Therefore, MYR could be developed into an effective drug to treat formaldehyde-induced carcinogenesis. | Li et al., 2022 |
| MYR promotes lung cancer cell death via ER stress pathway-induced pyroptosis. Hence, MYR could be effectively used as a pyroptosis agonist to further develop new anticancer drugs. | Han et al., 2022 |
| MYR has proved effective in normalizing the altered intestinal flora in mice affected by type 2 diabetes. | Zhao et al., 2022 |
| A solid lipid nanoparticle MYR formulation effectively leads to colon cancer cell death via increased reactive oxygen species (ROS) formation and promotion of the apoptotic process. | Alidadi et al., 2022 |
| MYR shows antidepressant and anxiolytic activity as a regulator of the hypothalamic-pituitary-adrenal axis and activator of the BDNF-extracellular signal-regulated kinase (ERK) signaling pathway. Hence, MYR may be helpful in the prevention of traumatic stress such as posttraumatic stress disorder. | Sur and Lee, 2022a |
| MYR successfully leads to apoptosis in hepatocellular carcinoma (HCC) cells by inducing ER stress. MYR also increases protective autophagy. Furthermore, inhibition of autophagy increases MYR's effectiveness against HCC. Therefore, MYR could become a valuable HCC treatment, particularly in combination with autophagy inhibitors. | Ji et al., 2022 |
| MYR effectively inhibits amyloid aggregation in α-synuclein condensates by delaying the liquid-to-solid phase transition. | Xu et al., 2022 |
| MYR, a pan-histone lysine demethylase family 4 (KDM4) inhibitor, is a potent cytotoxic agent against castration-resistant prostate cancer (CRPC) cells. In addition, the combination of poly lactic-co-glycolic acid (PLGA)-encapsulated MYR with enzalutamide is likely to be effective for CRPC. | Liu et al., 2022 |
| MYR inhibits PAR1-mediated epithelial-endothelial transition, while blocking HCC cell invasion, metastasis, vasculogenic mimicry formation and angiogenesis by binding Leu258 and Thr261 in the PAR1 receptor. | Wang et al., 2022 |
| Administering MYR can inhibit alcohol-induced hepatic injury via regulation of ethanol metabolism, reduction of the oxidative stress, control of the lipid profile, and suppression of inflammatory markers. | Ahmad et al., 2022 |
| MYR ameliorates 5-Fluorouracil-induced cardiac impairment by regulating inflammation, oxidation levels, and cardiac-specific markers. | Arafah et al., 2022 |
| MYR may reverse the mitochondrial impairment in N2a-SW cells, showing a potential neuroprotective activity for amyloid-precursor protein (APP)/amyloid-β (Aβ)-related illnesses, including AD. | Yao et al., 2022 |
| MYR acts as an antiviral against infectious bronchitis virus (IBV) by downregulating the deubiquitinating activity of the papain-like protease. As such, MYR could be a potential tool to prevent and treat IBV. | Peng et al., 2022 |
| MYR strongly blocks the proliferation of cancer cells MCF-7 and A549, also having an apoptotic effect. MYR could become a valid anticancer drug acting on microtubule-affinity regulating kinase-mediated diseases. | Anwar et al., 2022 |
| MYR displays antioxidant activity and the ability to inhibit the mitochondrial permeability transition pore, which eases aluminum phosphide-induced toxicity in isolated cardiomyocytes and mitochondria. These results suggest that it may be worth examining MYR's ***in vivo*** activity. | Salimi et al., 2021 |
| MYR is an orally available natural bruton tyrosine kinase inhibitor that effectively prevents lymphoma TMD-8 cell growth ***in vitro*** and ***in vivo***. Moreover, results from this study hint that MYR could be a promising novel drug for lymphoma treatment. | Song et al., 2021 |

EXCLI Journal 2023;22:1223-1231 – ISSN 1611-2156
Received: September 08, 2023, accepted: November 14, 2023, published: November 28, 2023

| Key findings | Reference |
|---|---|
| MYR has proved effective against nonalcoholic fatty liver disease via regulation of the expression of transcription factors of lipid metabolism in the liver, the antioxidant balance, and pro-inflammatory cytokines. | Choi et al., 2021 |
| MYR has an inhibiting effect on acute gastric damage caused by ethanol, which is exerted by limiting injury by oxidative stress, upregulating prostaglandin E2 production, and downregulating NF-κB activation. Therefore, MYR may be a novel approach to treat alcohol-induced gastric injury. | Park et al., 2021 |
| MYR exerts an anti-inflammatory and anti-EndMT effect on oxidized low-density lipoprotein (ox-LDL)-induced human umbilical vein endothelial cell (HUVEC) injury via regulation of GAS5/miR-29a-3p. Therefore, MYR could be useful for treating atherosclerosis. | Bai et al., 2021 |
| Flavonoids like MYR inhibit liquid-liquid phase separation and abnormal aggregation of Tau in neuronal cells. Results also show that MYR may be an autophagy-related protein 5 (ATG5)-dependent autophagic activator, and therefore a potential alternative treatment for AD. | Dai et al., 2021 |
| MYR inhibits neointimal hyperplasia and vascular smooth muscle cell expansion and migration by downregulating transforming growth factor-beta receptor 1 (TGFBR1) signaling. Therefore, MYR could become a new candidate drug to treat atherosclerosis and vascular restenosis. | Chen et al., 2021 |
| MYR alleviates ***Staphylococcus aureus'*** pathogenicity ***in vivo***, also aiding the effectiveness of the traditionally employed antibiotic oxacillin on methicillin-resistant S. aureus (MRSA) infection and preventing the death of mice from MRSA-induced fatal lung infections. Therefore, MYR may become a promising therapeutic agent against ***S. aureus***-induced illnesses. | Jing et al., 2021 |
| MYR decreases lipid synthesis and inflammation in the liver via modulation of fecal butyric-acid-related gut microbiota and protection of the gut barrier functionality. Such results could help understanding the action mechanism of low-bioavailability flavonoids. | Sun et al., 2021 |
| MYR exerts a potent activity on bleomycin-induced lung inflammation by limiting inflammatory cell infiltration and the secretion of inflammatory cytokines IL-6, IL-1α, TNF-α, and IFN-γ. MYR could therefore be useful to treat COVID-19. | Xiao et al., 2021 |
| MYR can greatly ameliorate mammary inflammation, probably due to its protective role exerted by inhibiting the phosphorylation level of P38 and ERK1/2 proteins. | Kan et al., 2021 |
| MYR prevents high-molecular-weight Aβ oligomers (HMW-Aβo)-induced neurotoxicity via a number of antioxidant activities. Therefore, MYR could be further developed into an effective AD drug. | Kimura et al., 2021 |
| MYR may protect natural killer cells from arsenite (As+3)-induced genetic damage by lowering oxidation levels and retaining poly (ADP-ribose) polymerase 1 (PARP-1) activity. This implies that MYR could prevent As+3-induced toxicity in NK cells. | Ma et al., 2021 |
| MYR can protect PC12 cells from cadmium-induced neurotoxicity, an activity related to MYR's antioxidant activity, suppression of lipid peroxidation, and inhibition of caspase-3 activation. | Aminzadeh and Salarinejad, 2021 |
| MYR, as a potential type III secretion system (T3SS) inhibitor, shows a protective activity both ***in vitro*** and ***in vivo***. As such, MYR has the potential to become a new antibiotic capable of treating ***S. typhimurium*** infections. | Lv et al., 2021 |
| MYR exerts a protective activity on rotenone-treated MES23.5 cells by strongly downregulating hepcidin expression which limits iron accumulation; this activity is related to a modification of STAT3 and SMAD1 signaling pathways. | Deng et al., 2021 |
| MYR shows cytotoxic activity by blocking cell cycle progression and promoting ROS-dependent mitochondria-facilitated death in A549 lung cancer cells. Therefore, MYR could be a potential drug candidate for lung cancer treatment. | Rajendran et al., 2021 |
| Molecular dynamics simulation studies hint that MYR acts on the human islet amyloid polypeptide (hIAPP) pentameric fibril model at the amyloidogenic core region, resulting in inhibited aggregation and distortion of the fibrils. | Dubey et al., 2020 |

EXCLI Journal 2023;22:1223-1231 – ISSN 1611-2156
Received: September 08, 2023, accepted: November 14, 2023, published: November 28, 2023

| Key findings | Reference |
|---|---|
| MYR induces apoptosis in T47D breast cancer cells by acting on extrinsic and intrinsic apoptotic pathways, probably by inducing the BRCA1-GADD45 pathway. | Soleimani and Sajedi, 2020 |
| MYR suppresses *S. aureus'* virulence by acting on Hla and lowering the inflammatory response in host cells. As such, together with traditional antibiotics, MYR could be a novel drug to treat *S. aureus* infections. | Wang et al., 2020 |
| MYR has the ability to trap methylglyoxal in mice. This suggests that administrating MYR-containing foods could lead to scavenging of MGO (methylglyoxal) ***in vivo*** and prevent MGO-induced dangerous effects on human health. | Zhang et al., 2020 |
| MYR activates apoptotic and autophagic processes in colon tumor cells, and for this reason it could be a valid candidate for chemotherapy, by inhibiting tumor growth alone or as an adjuvant agent to induce autophagy. | Zhu et al., 2020 |
| MYR may lower blood pressure either in the form of a dietary supplement or as a natural product suitable for the development of novel antihypertensive drugs. | Berköz et al., 2020 |
| MYR displays potent anti-schistosome activity both ***in vitro*** and ***in vivo***, and therefore could serve as the base for the development of a new therapeutic agent effective against ***S. japonicum***. | Huang et al., 2020 |
| MYR ameliorates myocardial damage and lethality in heat stroke via upregulation of the heat shock protein 72 and could be further developed into a novel agent to prevent heat stroke. | Lin et al., 2020 |
| MYR effectively inhibits A549-IR radioresistant lung cancer cell migration by blocking MMP-2 and MMP-9 expression via inhibition of the focal adhesion kinase (FAK)-ERK signaling pathway. | Kang et al., 2020 |
| The cellular uptake and antitumor activity of MYR in lung carcinoma are enhanced by nano encapsulated phospholipidic complexes. Therefore, a formulation of inhalable microparticles could become an effective therapy for lung carcinoma. | Nafee et al., 2020 |
| MYR exhibits great inhibitory activity against dipeptidyl peptidase-4 (DPP-4) both ***in vitro*** and ***in vivo***, resulting in increased circulating glucagon-like peptide 1 (GLP-1) and insulin levels, hence ameliorating diabetic symptomatology. However, even if MYR and horsegram protein individually ease the diabetic condition, their dietary combination shows a diminished efficiency. | Lalitha et al., 2020 |
| MYR exerts a protective effect on monoclonal gammopathy of unknown significance (MGUS) and its nutritional supplement may also protect against cancer development in patients affected by MGUS. | Akhtar et al., 2020 |
| A polyphenol-rich extract and its most abundant flavonoid MYR display strong inhibition of platelet function. Moreover, MYR has proved effective in inhibiting ERp5 and protein disulfide isomerase (PDI), therefore showing potential as a novel therapeutic agent for thrombotic disorders. | Gaspar et al., 2020 |
| MYR lowers BV2 microglial neuroinflammation via inhibition of the MAPK signaling pathway and the production of proinflammatory modulators and cytokines. Hence, MYR could be useful as a treatment for neuroinflammatory diseases. | Jang et al., 2020 |
| MYR can suppress amyloid formation: therefore, it could be further developed into new anti-amyloid drugs. | Prajapati et al., 2020 |
| MYR ameliorates streptozotocin-induced abnormal bone metabolism in rats and could therefore become a new therapeutic agent to treat diabetic bone disease. | Ying et al., 2020 |
| MYR has proved effective in easing inflammation in acute ulcerative colitis, greatly improving the condition. MYR induces higher expression of IL-10 and transforming growth factor β. Moreover, the number of regulatory T cells is noticeably higher in mice treated with MYR. | Qu et al., 2020 |
| MYR likely protects HUVECs from high glucose-induced oxidative stress by improving cell total antioxidant capacity and lowering Bax/Bcl-2 protein ratio, as well as lowering caspase-3 expression. | Aminzadeh and Bashiri, 2020 |

***Acknowledgments***

***Conflict of interest***

The authors declare no conflict of interest.

## Notes

Priscilla Nadalin and Jae Kwang Kim contributed equally as first author.

## Declaration

### Acknowledgments

This study was carried out with the support of 'R&D Program for Forest Science Technology (Project No. 2021379B10-2123-BD02)' provided by Korea Forest Service (Korea Forestry Promotion Institute).

### Conflict of interest

The authors declare no conflict of interest.

## Figures and Tables

**Table 1:**
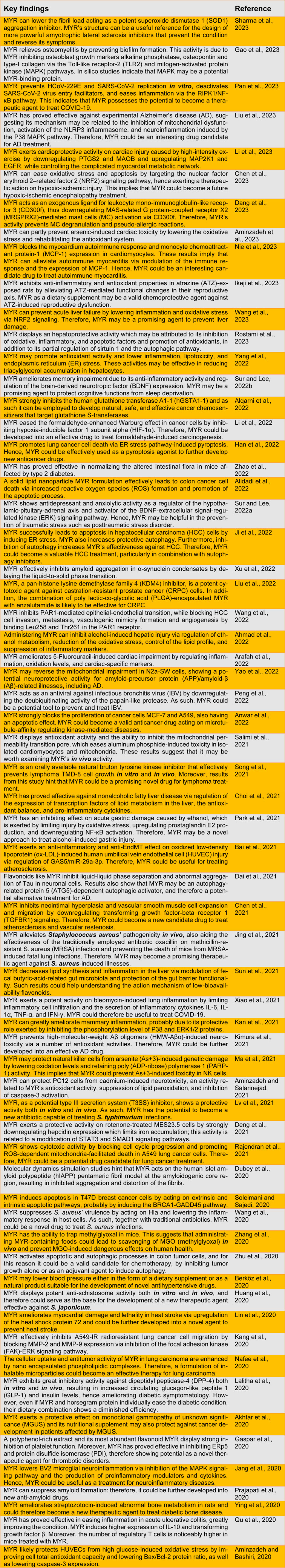
Recent research on the biological and pharmacological activities of myricetin

## References

[1] Agraharam G, Girigoswami A, Girigoswami K (2022). Myricetin: a multifunctional flavonol in biomedicine. Curr Pharmacol Rep.

[2] Ahmad SB, Rashid SM, Wali AF, Ali S, Rehman MU, Maqbool MT (2022). Myricetin (3, 3′, 4′, 5, 5′, 7-hexahydroxyflavone) prevents ethanol-induced biochemical and inflammatory damage in the liver of Wistar rats. Hum Exp Toxicol.

[3] Akhtar S, Najafzadeh M, Isreb M, Newton L, Gopalan RC, Anderson D (2020). An in vitro investigation into the protective and genotoxic effects of myricetin bulk and nano forms in lymphocytes of MGUS patients and healthy individuals. Toxicol Lett.

[4] Alidadi H, Ashtari A, Samimi A, Karami MA, Khorsandi L (2022). Myricetin loaded in solid lipid nanoparticles induces apoptosis in the HT-29 colorectal cancer cells via mitochondrial dysfunction. Mol Biol Rep.

[5] Alqarni MH, Foudah AI, Muharram MM, Alam A, Labrou NE (2022). Myricetin as a potential adjuvant in chemotherapy: studies on the inhibition of human glutathione transferase A1–1. Biomolecules.

[6] Aminzadeh A, Bashiri H (2020). Myricetin ameliorates high glucose‐induced endothelial dysfunction in human umbilical vein endothelial cells. Cell Biochem Funct.

[7] Aminzadeh A, Darijani MH, Bashiri H (2023). Investigating the effect of myricetin against arsenic-induced cardiac toxicity in rats. Toxicol Res (Camb).

[8] Aminzadeh A, Salarinejad A (2021). Effects of myricetin against cadmium-induced neurotoxicity in PC12 cells. Toxicol Res (Camb).

[9] Anwar S, Khan S, Anjum F, Shamsi A, Khan P, Fatima H (2022). Myricetin inhibits breast and lung cancer cells proliferation via inhibiting MARK4. J Cell Biochem.

[10] Arafah A, Rehman MU, Ahmad A, AlKharfy KM, Alqahtani S, Jan BL (2022). Myricetin (3, 3′, 4′, 5, 5′, 7-Hexahydroxyflavone) Prevents 5-Fluorouracil-Induced Cardiotoxicity. ACS Omega.

[11] Bai Y, Liu X, Chen Q, Chen T, Jiang N, Guo Z (2021). Myricetin ameliorates ox-LDL-induced HUVECs apoptosis and inflammation via lncRNA GAS5 upregulating the expression of miR-29a-3p. Sci Rep.

[12] Berköz M, Yıldırım M, Yalın S, İlhan M, Yunusoğlu O (2020). Myricetin inhibits angiotensin converting enzyme and induces nitric oxide production in HUVEC cell line. Gen Physiol Biophys.

[13] Chen G, Xu H, Wu Y, Han X, Xie L, Zhang G (2021). Myricetin suppresses the proliferation and migration of vascular smooth muscle cells and inhibits neointimal hyperplasia via suppressing TGFBR1 signaling pathways. Phytomedicine.

[14] Chen T, Hu Y, Lu L, Zhao Q, Tao X, Ding B (2023). Myricetin attenuates hypoxic-ischemic brain damage in neonatal rats via NRF2 signaling pathway. Front Pharmacol.

[15] Choi HN, Shin JY, Kim JI (2021). Ameliorative effect of myricetin on nonalcoholic fatty liver disease in ob/ob mice. J Med Food.

[16] Dai B, Zhong T, Chen ZX, Chen W, Zhang N, Liu XL (2021). Myricetin slows liquid–liquid phase separation of tau and activates ATG5-dependent autophagy to suppress tau toxicity. J Biol Chem.

[17] Dang B, Hu S, Zhang Y, Huang Y, Zhang T, An H (2023). Myricetin served as antagonist for negatively regulate MRGPRX2 mediated pseudo-allergic reactions through CD300f/SHP1/SHP2 phosphorylation. Int Immunopharmacol.

[18] Deng H, Liu S, Pan D, Jia Y, Ma ZG (2021). Myricetin reduces cytotoxicity by suppressing hepcidin expression in MES23. 5 cells. Neural Regen Res.

[19] Dias MC, Pinto DC, Silva AM (2021). Plant flavonoids: Chemical characteristics and biological activity. Molecules.

[20] Dubey R, Kulkarni SH, Dantu SC, Panigrahi R, Sardesai DM, Malik N (2020). Myricetin protects pancreatic β-cells from human islet amyloid polypeptide (hIAPP) induced cytotoxicity and restores islet function. Biol Chem.

[21] Flamini R, Mattivi F, De Rosso M, Arapitsas P, Bavaresco L (2013). Advanced knowledge of three important classes of grape phenolics: anthocyanins, stilbenes and flavonols. Int J Mol Sci.

[22] Gao L, Tang Z, Li T, Wang J (2023). Myricetin exerts anti-biofilm activity and attenuates osteomyelitis by inhibiting the TLR2/MAPK pathway in experimental mice. Microb Pathog.

[23] Gaspar RS, da Silva SA, Stapleton J, Fontelles JLdL, Sousa HR, Chagas VT (2020). Myricetin, the main flavonoid in Syzygium cumini leaf, is a novel inhibitor of platelet thiol isomerases PDI and ERp5. Front Pharmacol.

[24] Gupta G, Siddiqui MA, Khan MM, Ajmal M, Ahsan R, Rahaman MA (2020). Current pharmacological trends on myricetin. Drug Res (Stuttg).

[25] Han J, Cheng C, Zhang J, Fang J, Yao W, Zhu Y (2022). Myricetin activates the Caspase-3/GSDME pathway via ER stress induction of pyroptosis in lung cancer cells. Front Pharmacol.

[26] Huang P, Zhou M, Cheng S, Hu Y, Gao M, Ma Y (2020). Myricetin possesses anthelmintic activity and attenuates hepatic fibrosis via modulating TGFβ1 and Akt signaling and shifting Th1/Th2 balance in Schistosoma japonicum-infected mice. Front Immunol.

[27] Ikeji CN, Adedara IA, Farombi EO (2023). Dietary myricetin assuages atrazine-mediated hypothalamic-pituitary–testicular axis dysfunction in rats. Environ Sci Pollut Res Int.

[28] Imran M, Saeed F, Hussain G, Imran A, Mehmood Z, Gondal TA (2021). Myricetin: A comprehensive review on its biological potentials. Food Sci Nutr.

[29] Jang JH, Lee SH, Jung K, Yoo H, Park G (2020). Inhibitory effects of myricetin on lipopolysaccharide-induced neuroinflammation. Brain Sci.

[30] Javed Z, Khan K, Herrera-Bravo J, Naeem S, Iqbal MJ, Raza Q (2022). Myricetin: targeting signaling networks in cancer and its implication in chemotherapy. Cancer Cell Int.

[31] Ji A, Hu L, Ma D, Qiang G, Yan D, Zhang G (2022). Myricetin induces apoptosis and protective autophagy through endoplasmic reticulum stress in hepatocellular carcinoma. Evid Based Complement Alternat Med.

[32] Jing S, Wang L, Wang T, Fan L, Chen L, Xiang H (2021). Myricetin protects mice against MRSA-related lethal pneumonia by targeting ClpP. Biochem Pharmacol.

[33] Kan X, Liu J, Chen Y, Guo W, Xu D, Cheng J (2021). Protective effect of myricetin on LPS-induced mastitis in mice through ERK1/2 and p38 protein author. Naunyn Schmiedebergs Arch Pharmacol.

[34] Kang HR, Moon JY, Ediriweera MK, Song YW, Cho M, Kasiviswanathan D (2020). Dietary flavonoid myricetin inhibits invasion and migration of radioresistant lung cancer cells (A549‐IR) by suppressing MMP‐2 and MMP‐9 expressions through inhibition of the FAK‐ERK signaling pathway. Food Sci Nutr.

[35] Kim JD, Liu L, Guo W, Meydani M (2006). Chemical structure of flavonols in relation to modulation of angiogenesis and immune-endothelial cell adhesion. J Nutr Biochem.

[36] Kimura AM, Tsuji M, Yasumoto T, Mori Y, Oguchi T, Tsuji Y (2021). Myricetin prevents high molecular weight Aβ1-42 oligomer-induced neurotoxicity through antioxidant effects in cell membranes and mitochondria. Free Radic Biol Med.

[37] Kumar S, Pandey AK (2013). Chemistry and biological activities of flavonoids: an overview. ScientificWorldJournal.

[38] Lalitha N, Sadashivaiah B, Ramaprasad TR, Singh SA (2020). Anti-hyperglycemic activity of myricetin, through inhibition of DPP-4 and enhanced GLP-1 levels, is attenuated by co-ingestion with lectin-rich protein. PloS One.

[39] Li L, Ma H, Li D, Shu Q, Wang T, Song X (2022). Myricetin alleviates the formaldehyde-enhanced Warburg effect in tumor cells through inhibition of HIF-1α. Toxicol Appl Pharmacol.

[40] Li T, Wang L, Wu L, Xie Y, Chang M, Wang D (2023). Integrated metabolomics and network pharmacology investigation of cardioprotective effects of myricetin after 1-week high-intensity exercise. Nutrients.

[41] Lin X, Lin CH, Liu R, Li C, Jiao S, Yi X (2020). Myricetin against myocardial injury in rat heat stroke model. Biomed Pharmacother.

[42] Liu JS, Fang WK, Yang SM, Wu MC, Chen TJ, Chen CM (2022). Natural product myricetin is a pan-KDM4 inhibitor which with poly lactic-co-glycolic acid formulation effectively targets castration-resistant prostate cancer. J Biomed Sci.

[43] Liu P, Zhou Y, Shi J, Wang F, Yang X, Zheng X (2023). Myricetin improves pathological changes in 3× Tg-AD mice by regulating the mitochondria-NLRP3 inflammasome-microglia channel by targeting P38 MAPK signaling pathway. Phytomedicine.

[44] Lv Q, Lv Y, Dou X, Wassy SL, Jia G, Wei L (2021). Myricetin inhibits the type III secretion system of Salmonella enterica serovar typhimurium by downregulating the Salmonella pathogenic island I gene regulatory pathway. Microb Pathog.

[45] Ma H, Song X, Huang P, Zhang W, Ling X, Yang X (2021). Myricetin protects natural killer cells from arsenite induced DNA damage by attenuating oxidative stress and retaining poly (ADP-Ribose) polymerase 1 activity. Mutat Res Genet Toxicol Environ Mutagen.

[46] Nafee N, Gaber DM, Elzoghby AO, Helmy MW, Abdallah OY (2020). Promoted antitumor activity of myricetin against lung carcinoma via nanoencapsulated phospholipid complex in respirable microparticles. Pharm Res.

[47] Nie N, Li Z, Li W, Huang X, Jiang Z, Shen Y (2023). Myricetin ameliorates experimental autoimmune myocarditis in mice by modulating immune response and inhibiting MCP-1 expression. Eur J Pharmacol.

[48] Ong KC, Khoo HE (1997). Biological effects of myricetin. Gen Pharmacol.

[49] Pan H, He J, Yang Z, Yao X, Zhang H, Li R (2023). Myricetin possesses the potency against SARS-CoV-2 infection through blocking viral-entry facilitators and suppressing inflammation in rats and mice. Phytomedicine.

[50] Park HS, Seo CS, Baek EB, Rho JH, Won YS, Kwun HJ (2021). Gastroprotective effect of myricetin on ethanol-induced acute gastric injury in rats. Evid Based Complement Alternat Med.

[51] Peng S, Fang C, He H, Song X, Zhao X, Zou Y (2022). Myricetin exerts its antiviral activity against infectious bronchitis virus by inhibiting the deubiquitinating activity of papain-like protease. Poult Sci.

[52] Pluta R, Januszewski S, Czuczwar SJ (2021). Myricetin as a promising molecule for the treatment of post-ischemic brain neurodegeneration. Nutrients.

[53] Prajapati KP, Singh AP, Dubey K, Ansari M, Temgire M, Anand BG (2020). Myricetin inhibits amyloid fibril formation of globular proteins by stabilizing the native structures. Colloids Surf B Biointerfaces.

[54] Qu X, Li Q, Song Y, Xue A, Liu Y, Qi D (2020). Potential of myricetin to restore the immune balance in dextran sulfate sodium-induced acute murine ulcerative colitis. J Pharm Pharmacol.

[55] Rajendran P, Maheshwari U, Muthukrishnan A, Muthuswamy R, Anand K, Ravindran B (2021). Myricetin: Versatile plant based flavonoid for cancer treatment by inducing cell cycle arrest and ROS–reliant mitochondria-facilitated apoptosis in A549 lung cancer cells and in silico prediction. Mol Cell Biochem.

[56] Rostami A, Baluchnejadmojarad T, Roghani M (2023). Hepatoprotective effect of myricetin following lipopolysaccharide/DGalactosamine: Involvement of autophagy and sirtuin 1. Curr Mol Pharmacol.

[57] Salimi A, Jamali Z, Shabani M (2021). Antioxidant potential and inhibition of mitochondrial permeability transition pore by myricetin reduces aluminium phosphide-induced cytotoxicity and mitochondrial impairments. Front Pharmacol.

[58] Sharma S, Tomar VR, Deep S (2023). Myricetin: A potent anti-amyloidogenic polyphenol against superoxide dismutase 1 aggregation. ACS Chem Neurosci.

[59] Soleimani M, Sajedi N (2020). Myricetin apoptotic effects on T47D breast cancer cells is a P53-independent approach. Asian Pac J Cancer Prev.

[60] Song X, Rao H, Guo C, Yang B, Ren Y, Wang M (2021). Myricetin exhibit selective anti-lymphoma activity by targeting BTK and is effective via oral administration in vivo. Phytomedicine.

[61] Spiegel M, Andruniów T, Sroka Z (2020). Flavones’ and flavonols’ antiradical structure–activity relationship—A quantum chemical study. Antioxidants (Basel).

[62] Sun WL, Li XY, Dou HY, Wang XD, Li JD, Shen L (2021). Myricetin supplementation decreases hepatic lipid synthesis and inflammation by modulating gut microbiota. Cell Rep.

[63] Sur B, Lee B (2022). Myricetin Inhibited fear and anxiety-like behaviors by HPA axis regulation and activation of the BDNF-ERK signaling pathway in posttraumatic stress disorder rats. Evid Based Complement Alternat Med.

[64] Sur B, Lee B (2022). Myricetin prevents sleep deprivation-induced cognitive impairment and neuroinflammation in rat brain via regulation of brain-derived neurotropic factor. Korean J Physiol Pharmacol.

[65] Taheri Y, Suleria HAR, Martins N, Sytar O, Beyatli A, Yeskaliyeva B (2020). Myricetin bioactive effects: Moving from preclinical evidence to potential clinical applications. BMC Complement Med Ther.

[66] Wang M, Ren S, Bi Z, Zhang L, Cui M, Sun R (2022). Myricetin reverses epithelial–endothelial transition and inhibits vasculogenic mimicry and angiogenesis of hepatocellular carcinoma by directly targeting PAR1. Phytother Res.

[67] Wang T, Zhang P, Lv H, Deng X, Wang J (2020). A natural dietary flavone myricetin as an α-hemolysin inhibitor for controlling Staphylococcus aureus infection. Front Cell Infect Microbiol.

[68] Wang X, Sun Y, Li P, Wu Z, Chen Y, Fu Y (2023). The protective effects of myricetin against acute liver failure via inhibiting inflammation and regulating oxidative stress via Nrf2 signaling. Nat Prod Res.

[69] Xiao T, Cui M, Zheng C, Wang M, Sun R, Gao D (2021). Myricetin inhibits SARS-CoV-2 viral replication by targeting Mpro and ameliorates pulmonary inflammation. Front Pharmacol.

[70] Xu B, Mo X, Chen J, Yu H, Liu Y (2022). Myricetin inhibits α‐synuclein amyloid aggregation by delaying the liquid‐to‐solid phase transition. Chembiochem.

[71] Yang W, Yang M, Tian Y, Jiang Q, Loor JJ, Cao J (2022). Effect of myricetin on lipid metabolism in primary calf hepatocytes challenged with long-chain fatty acids. Metabolites.

[72] Yao X, Zhang J, Lu Y, Deng Y, Zhao R, Xiao S (2022). Myricetin restores Aβ-induced mitochondrial impairments in N2a-SW cells. ACS Chem Neurosci.

[73] Ying X, Chen X, Wang T, Zheng W, Chen L, Xu Y (2020). Possible osteoprotective effects of myricetin in STZ induced diabetic osteoporosis in rats. Eur J Pharmacol.

[74] Zhang S, Xiao L, Lv L, Sang S (2020). Trapping methylglyoxal by myricetin and its metabolites in mice. J Agric Food Chem.

[75] Zhao Z, Chen Y, Li X, Zhu L, Wang X, Li L (2022). Myricetin relieves the symptoms of type 2 diabetes mice and regulates intestinal microflora. Biomed Pharmacother.

[76] Zhu Ml, Zhang PM, Jiang M, Yu SW, Wang L (2020). Myricetin induces apoptosis and autophagy by inhibiting PI3K/Akt/mTOR signalling in human colon cancer cells. BMC Complement Med Ther.

